# Polymedication Electronic Monitoring System (POEMS) – a new technology for measuring adherence

**DOI:** 10.3389/fphar.2013.00026

**Published:** 2013-03-13

**Authors:** Isabelle Arnet, Philipp N. Walter, Kurt E. Hersberger

**Affiliations:** Pharmaceutical Care Research Group, Department of Pharmaceutical Sciences, University of BaselBasel, Switzerland

**Keywords:** compliance, adherence, electronic monitoring, multidrug punch card, printed electronics, community pharmacy

## Abstract

**Introduction:** Reliable and precise measurement of patient adherence to medications is feasible by incorporating a microcircuitry into pharmaceutical packages of various designs, such that the maneuvers needed to remove a dose of drug are detected, time-stamped, and stored. The principle is called “electronic medication event monitoring” but is currently limited to the monitoring of a single drug therapy.

**Aim:** Our aims were introducing a new technology; a clear, self-adhesive polymer film, with printed loops of conductive wires that can be affixed to multidrug punch cards for the electronic adherence monitoring of multiple medication regimens (Polymedication Electronic Monitoring System, POEMS), and illustrating potential benefits for patient care. We present a preliminary report with one patient experience.

**Materials and methods:** Our illustrative case was supplied with a pre-filled 7-day multiple medication punch card with unit-of-use doses for specific times of the day (six pills in the morning cavity, two pills in the evening cavity, and one pill in case of insomnia in the bedtime cavity), with the new electronic film affixed on it.

**Results:** The intake times over 1 week were extremely skewed (median intake hours at 2:00 pm for the morning doses and at 6:40 pm for the evening doses). After an intervention aimed at optimizing the timing adherence, the morning and evening intake hours became more balanced, with 42.3% of correct dosing intervals (±3 h) for drugs with twice daily intake (vs. 0% before the intervention).

**Discussion:** The electronic monitoring of the entire therapy revealed an intake pattern that would have remained undiscovered with any other device and allowed a personalized intervention to correct an inadequate medication intake behavior. POEMS may guide health professionals when they need to optimize a pharmacotherapy because of suspected insufficient adherence. Further, knowing the intake pattern of the entire pharmacotherapy can elucidate unreached clinical outcome, drug–drug interactions, and drug resistance. In the near future, one could imagine that medication adherence data over the entire therapy plan would be available as soon as the electronic wires are activated, so that a failure to take medication could be detected immediately and intervention could be taken if appropriate.

## INTRODUCTION

The ideal measurement of adherence has long since been described (Rudd, 1979; Pullar and Feely, 1990) and should be non-invasive, unobtrusive (to avoid that the drug-taking behavior of the patient is influenced by the device), objective (to generate reproducible data for each subject), reliable (to insure that the prescribed dose was really taken at the time of package opening), practical and cheap (to maximize use and minimize costs). It should also yield immediate results and not be open to manipulation. Based on these stringent requirements, traditional, indirect measures (i.e., which do not demonstrate drug ingestion, such as self-reporting, medication diaries, residual pill counting, pharmacy records, and clinician opinion) do satisfy many criteria (Laufs et al., 2011). However, they assume rather than prove the patient’s actual drug intake, albeit that they cover longer periods of time. On the contrary, direct methods (i.e., detection of the drug or a metabolic product in a biologic fluid) prove that a dose of a drug was taken but cover brief medication periods. With the emergence of microprocessor technologies in the 1990s, the precise timing of medication-taking behavior with oral solid forms became feasible, and revealed a comprehensive picture of an individual’s day-to-day drug intake that neither drug serum concentrations nor pill counts would have identified. Although electronic compliance-monitoring devices (ECMD) are considered to provide the most accurate and valuable data (Hughes, 2007) and are close to a “gold standard” in measuring adherence, they have been mainly used until now as a research tool, owing to their prohibitive cost. Electronic monitoring is used in research areas to measure adherence in population or in clinical studies; to assess determinants of adherence, and to evaluate the effects of intervention on adherence. On the patient level, electronic monitoring allows to calculate dosing intervals, taking and timing adherence; to identify specific patterns of medication use including week-end effects, drug holidays (discontinuing medication use for 24–72 h), toothbrush effect or white-coat adherence (increasing adherence several days prior to a medical appointment), and dumping (intentionally discarding medication); to identify days of under- and over-consumption; to link the timing of doses with the efficacy of the drug and with critical health incident (Riekert and Rand, 2002); to distinguish between probable and improbable drug reactions or side effects (Rudd, 1993; Riekert and Rand, 2002), and finally to give patients insight into their own dosing history. The ECMD use a microprocessor embedded in a pill bottle cap or in a storage container (Haberer et al., 2012), that records the precise date and time, every instance that the device is opened and closed. The major drawback of the bottle is that it monitors only one lead drug and thus requires one cap per medication, while the container holds up to 1-month supply of different pills in its five inner compartments. Due to this setting, data are missing on what was done at each opening; was it to take one or more pills, to remove daily pocket doses or to fill a weekly organizer? (Samet et al., 2001). Further, both devices do not accommodate the use of pillboxes (Hayes et al., 2006).

**FIGURE 1:**
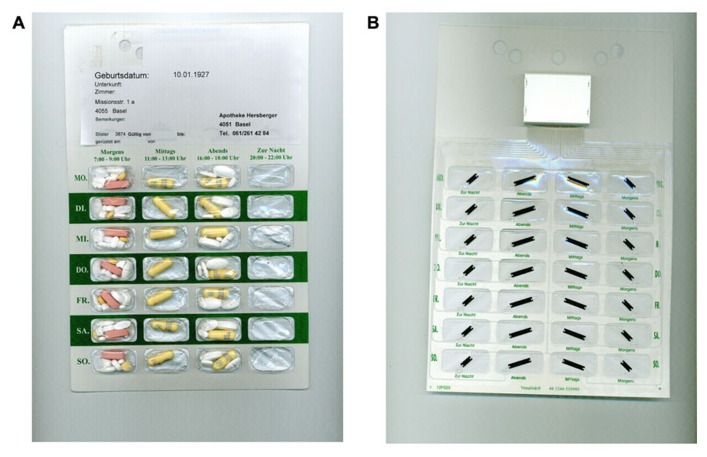
**(A)** Front side of a commercially available multidose punch card (Pharmis GmbH, Beinwil am See, Switzerland) with 7×4 cavities pre-filled with a patient’s individualized medication regimen. **(B)** Rear side covered with an electronic film of conductive tracks, a battery and an antenna, and a microchip housing (Confrérie Clinique S.A., Lausanne, Switzerland).

The new technology is composed of printed electronics made of a clear, self-adhesive polymer film with loops of conductive wires that can be affixed to blister packagings. The smart components measure the electrical resistance and record the time of its changes when a loop is broken, i.e., when a cavity is emptied. The data are transferred via a wireless communication device to a web-based database. This new technology was first developed to fit commercially available standard blister packs (Jekle and Krämer, 2008), avoiding the transfer of pills into an ECMD and keeping the primary packaging. We developed further the electronic film technology to fit on the rear side of a disposable multidose punch card (**Figure 1**). This “unit-of-use packaging” consists of sealed calendar compartments with several medications to be taken together in fixed combination, thus avoiding patients from having to use multiple medication packs and bottles. Currently, multidose punch cards are filled manually by a host of community pharmacists, e.g., in the UK, Switzerland, Germany, France, Canada, and Australia. With the electronic film applied to a multidose punch card, an individualized polytherapy can be monitored by means of the so-called Polymedication Electronic Monitoring System (POEMS).

The purpose of this paper is to present an illustrative case using a new technology of electronic adherence measurement of multiple medication regimens with oral solid forms, and to estimate the possible implications linked to this novel technology. We present a preliminary report with one patient experience.

## MATERIALS AND METHODS

### EXTENDED CASE REPORT

Our patient is a single, recently retired, 65-year-old, Caucasian male. He lives independently (and alone) in an apartment in a medium-sized Swiss city and is in possession of a valid driver’s license. He did have a history of alcohol abuse 20 years previously, which he was able to overcome. Epilepsy was diagnosed in 1974 and is currently controlled with levetiracetam 1000 mg twice daily. He is prescribed paroxetine 20 mg once daily for the treatment of social phobia and relapsing depression. Persisting, slightly asymptomatic anemia has been repeatedly investigated without conclusive diagnosis. Probationary treatment with a vitamin B complex twice daily since October 2010 led to a partial correction of the anemia. Rosuvastatin 20 mg and low-dose Aspirin^®^ 100 mg were prescribed once daily for secondary prevention after a cardiovascular incident. Hypothyroidism was picked up in March 2011 and is being treated with levothyroxine 0.1 mg once daily. Zolpidem 10 mg once daily is being taken when required for difficulty sleeping. The patient was briefly hospitalized in May 2010 for breakthrough seizures. His physician was suspecting non-adherence with anti-epileptic drugs, while his pharmacist suspected an over-consumption of sleeping pills because the patient would regularly come between the regular refill times, requiring additional zolpidem tablets. Since hospital discharge, the patient was using a pill organizer, refilled weekly by his community pharmacist.

The patient was offered by his physician in August 2010 to get his medication intake monitored, and he accepted. The pharmacist repackaged the entire regimen in a weekly 7×4-cavities punch card with POEMS, with six pills in the morning cavity (levothyroxine, rosuvastatin, Aspirin^°ledR^, paroxetine, vitamin B complex, and levetiracetam), two pills in the evening cavity (vitamin B complex and levetiracetam) and one pill for sleep disorder in the bedtime cavity (zolpidem). The noon cavity was left emptied. The remainder of the patient’s monthly medication was stored at the study center to ensure that no other medication would be taken beside that prescribed and individually blistered. The patient was informed of the electronic monitoring system and was advised to take his drugs as instructed by his physician.

The following parameters were derived from the electronic reports, where “dose” is defined as “unit-of-use drugs” included in one cavity, according to the therapy plan.

(a) Percentage overall taking adherence (total doses taken divided by total number of prescribed doses) calculated over the duration of the observational period;
(b) Percentage of correct dosing days (days taking prescribed dose divided by total days of prescribed dose) calculated over the duration of the observational period;
(c) Percentage of correct dosing intervals (number of correct dosing intervals divided by total number of prescribed dosing intervals) calculated over the duration of the observational period; a dosing interval is defined as correct if the time between doses is within 25% of the prescribed dosing interval (±6 h for a 24-h period and ±3 h for a 12-h period).

## RESULTS

**FIGURE 2:**
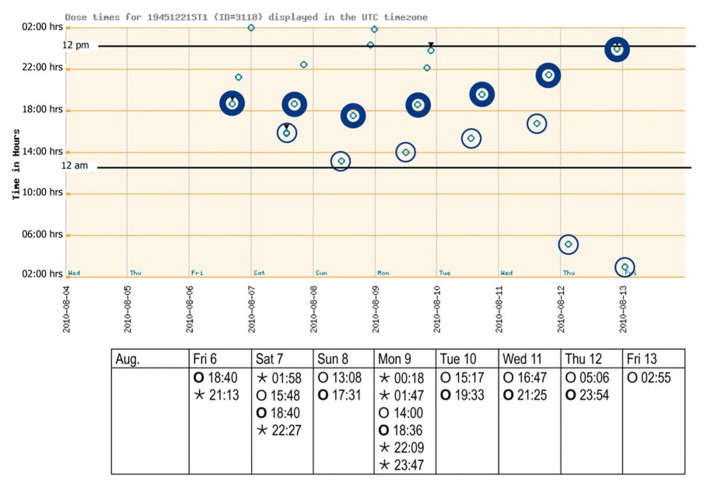
**Adherence report over 1 week after inclusion (August 2010).** The electronic punch card was handed out on Friday morning, with the first cavity to be opened on the Friday evening. The spots (see graph) reflect a pushing through of all drugs contained in one distinct cavity as recorded with date and time (see table) by the electronic wires in the film. Morning and evening doses are highlighted. Bedtime doses could be taken when needed. Key: ⊚ ○, morning doses; evening doses; º ⋆, bedtime doses.

Laboratory data at baseline showed no abnormalities beside a mild normochromic and normocytic anemia [hemoglobin 132 g/l (normal 140–180 g/l); red blood cells 4.35 T/l (normal 4.5–5.5 T/l)]. The very low cholesterol level [2.9 mmol/l (normal <5.0 mmol/l)] suggested that the patient was taking his lipid lowering agent well. The first weekly report of the monitored pill intake is given in **Figure 2**. The patient started his daily activities around noon. Median intake hours, mean intervals between doses, and adherence parameters are given in **Table 1**. As intervals between morning and evening doses were skewed compared to the theoretical 12-h dose interval for a twice daily intake, the percentage of correct dosing intervals for drugs contained in morning and evening doses, such as levetiracetam (intake ±3 h every 12 h) was 0%. The opening times of the bedtime cavities containing the sleeping pills showed a doubling of the dose during the first days of the week, leaving the patient without sleeping pills for the rest of the week.

**Table 1:** Intake times, intervals between doses, and adherence parameters for the two periods of adherence monitoring before (August 2010) and after (December 2010) the individualized intervention

	Before intervention (7 days in August 2010)	After intervention (14 days in December 2010)
Time of intake in the morning 8:00 am (median; interquartile range)	2:00 pm (10 h 42 min)	5:29 am (6 h 08 min)
Time of intake in the evening 8 pm (median; interquartile range)	6:40 pm (2 h 49 min)	7:09 pm (2 h 38 min)
Intervals between morning doses (mean ± SD)	21 h 51 min ± 5 h	23 h 53 min ± 7 h 31 min
Intervals between evening doses (mean ± SD)	24 h 50 min ± 1 h 15 min	24 h 03 min ± 3 h 10 min
Intervals between morning and evening doses (mean ± SD)	6 h 57 min ± 6 h 34 min	11 h 28 min ± 4 h 28 min
Intervals between evening and morning doses (mean ± SD)	16 h 07 min ± 7 h 28 min	12 h 25 min ± 5 h 59 min
Overall taking compliance	100%	102.5
Correct dosing days*	100%	100%
Correct dosing intervals morning, e.g., acetylsalicylic acid (24 ± 6 h)	83.3%	53.8%
Correct dosing intervals morning and evening, e.g., levetiracetam (12 ± 3 h)	0%	42.3%

* Without optional bedtime doses.

A measurement-guided medication management (MGMM) program (Hughes, 2007) was implemented by the physician after viewing the records of the polymedication adherence monitoring. Providing patients with feedback of their dosing histories has been shown to positively modify adherence behavior, either with cue-dose training (Rosen et al., 2004) or by raising awareness of the implications of current behavior (de Bruin et al., 2005). Thus, an intervention using elements of the ACE-ME model (assessment, collaboration, education, monitoring, and evaluation; Gould and Mitty, 2010) was planned with the pharmacist. The method of motivational interviewing (Miller and Rollnick, 2009) should be used by the pharmacist, i.e., open-ended questions, reflective listening, affirmation, and summarization to help the patient express his concerns about the behavioral change, enhance his personal motivation, set goals and arrive at a change of plan. The planned intervention should focus on the distorted dosing intervals. The objective of the intervention should be the improving of the patient’s timing adherence. After the final preparations were made, a session of 2 h was scheduled for the intervention and took place at the community pharmacy on Thursday, December 16, 2010. The reports of the intake pattern were printed out and discussed with the patient. The patient was instructed that paroxetine needs to be taken in the morning because of possible activating side effects, such as nervousness or difficulty sleeping, which are undesirable in the evening. A second aspect was the twice daily intake of the immediate release tablets: levetiracetam. The pharmacist explained that intake 12 h apart would result in constant plasma concentrations, whilst minimizing concentration-related adverse effects, such as hostility/aggression, anxiety, insomnia, and nervousness/irritability (European Medicine Agency (EMA), 2009). The patient should start on the next day morning with the new intake behavior he agreed on.

**FIGURE 3:**
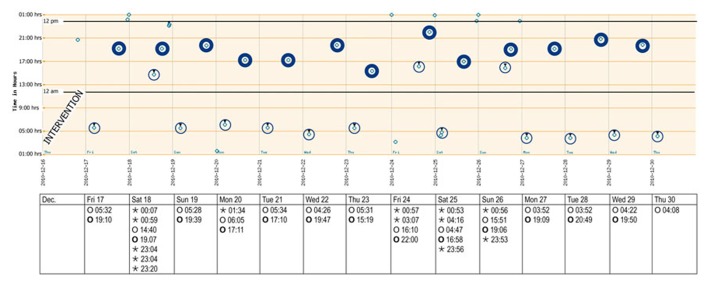
**Adherence report over 2 weeks after the intervention (December 2010).**The *y*-axis reflects local time after adjusting for wintertime (-1 h). A punch card was handed out every Thursday afternoon, with the first cavity to be opened on the Friday morning. Morning and evening doses are highlighted. Bedtime doses could be taken when needed. Key: ⊚ ○, morning doses;, evening doses; º ⋆, bedtime doses.

The records of the next 14 days subsequent to intervention are shown in **Figure 3**. A punch card was handed out every Thursday afternoon, with the first cavity to be opened on the Friday morning. The last visit was scheduled for the morning of Thursday, December 30. Overall taking adherence after intervention was 102.5% due to the anticipated consumption of sleeping pills before the last visit (**Table 1**). Time lapse between the 14 morning doses was close to the theoretical 24 h. The morning–evening and evening–morning intervals were close to 12 h and showed a higher constancy than before the intervention. As a consequence, the percentage of correct dosing intervals for drugs contained in morning and evening doses, such as levetiracetam (every 12 ± 3 h) reached 42.3% compared to 0% before intervention. The physician received the records, discussed them with the patient at the next visit, prescribed a double-dose of the sleeping pills and planed another session with the pharmacist aimed at motivating further the patient to persist in keeping his new intake pattern.

## DISCUSSION

We present a new and innovative film technology for monitoring adherence to multiple medication by means of a single case of a patient implementing a complex dosing regimen. To the best of our knowledge, this is the first time that drug intake patterns of an entire pharmacotherapy, scheduled at 8:00 am, 8:00 pm, and bedtime, have been monitored accurately and objectively in real-time. The problems suspected over months by the treating physician and the community pharmacist in the reported case (seizures due to insufficient adherence, over-consumption of sleeping pills) could not be solved satisfactorily with the measures then at disposition, like dispensing the medication in a pillbox. Only the electronic monitoring of the entire pharmacotherapy revealed the irregular pattern of the medication intake and the selective consumption of sleeping pills. The pattern would have remained undiscovered if only one lead drug had been tracked, e.g., with an electronic pill cap; and even unsuspected if the tracked drug had to be taken in the evening (mean interval between evening doses: 24 h 50 min). A personalized and targeted intervention could only be set up after the health professionals were aware of the distorted medication use. Thus, POEMS could guide health professionals when they optimize the treatment of patients whose unsatisfactory clinical outcome is suspected to depend on insufficient adherence behavior. This new technology could thus find its place in ambulatory care, e.g., in specific patients when physicians suspect any form of deviant adherence, as well as in clinical trials, e.g., with critical drugs or expensive drugs, when non-adherence must be excluded with strong certainty. The actual costs of the multidose punch cards are low (around €2 for one punch card), and the Swiss health insurance reimburse the adherence aid delivered by a community pharmacist as a cognitive service. The electronic film as research prototypes are at a high price, that will decrease as soon as the production can be automated, and reach an affordable price.

One limitation inherent to the electronic monitoring of medication use is that the patient gets no other medication than the individually repackaged drugs, in order to prevent any extra medication intake that would not be recorded. The lack of medication stock as well as the obligation to have punctual refills might be a constraint too strong for some patients and might represent a selection bias in larger studies. However, some patients welcome the simplification obtained with one multidrug punch card and the suppression of the different primary packagings. Further, some patients may be reluctant to use this technology because they may feel under surveillance. However, when the monitoring is not presented as a supervision but as a way to treatment optimization, one can suppose that the patients will accept an electronic monitoring. We observed also a marked curiosity from our patient as well as a certain desire to compete with the technique.

When searching for a gold standard for adherence monitoring, electronic films affixed to multiple medication punch cards appear to fill all the criteria, i.e., they are non-invasive, unobtrusive, objective, and user friendly. In addition, the transparent compartments on the front side facilitate visual verification of the pre-filled medication and contribute to the safety of drug intake. The monitoring of a multiple drug regimen depicts the intake times of all drugs and thus, enables to evaluate complex drug–effect relationship like drug resistance and drug–drug interactions. Finally, the new system is usable, even when a patient is used to storage devices like a pillbox.

Some studies showed that short message services (SMS) sent automatically to patients at the appropriate time without interference of a healthcare professional have positive effects on adherence rate (Vervloet et al., 2012). Further, first results with transmission of adherence data through telephone connection in real-time showed the feasibility of the immediate monitoring and its potential to give feedback when a dose of a drug is not taken. Thus, in the near future, one could imagine that medication adherence data over the entire therapy plan would be available as soon as the electronic wires are activated, so that a failure to take medication could be detected immediately and intervention could be taken if appropriate, like sending a SMS reminder. We are well aware that we present a single case to depict new emerging fields of monitoring a polymedication. Further studies are needed to confirm the generalizability of our findings and to establish the place of POEMS in ambulatory care and in clinical trials.

## Conflict of Interest Statement

The authors declare that the research was conducted in the absence of any commercial or financial relationships that could be construed as a potential conflict of interest.
